# Giant Zeeman-type spin splitting of free electron/hole states on quasi-2D perovskite niobates: a theoretical prediction

**DOI:** 10.1038/s41598-020-60653-8

**Published:** 2020-02-28

**Authors:** Yi Zhou, Fei Zhou, Yong Liu, Zhonghong Lai, Mingqing Liao, Yudong Huang, Jingchuan Zhu

**Affiliations:** 1 School of Materials Science and Engineering, Harbin Institute of Technology, 150001 Harbin, China; 2 MIIT Key Laboratory of Critical Materials Technology for New Energy Conversion and Storage, School of Chemistry and Chemical Engineering, Harbin Institute of Technology, 150001 Harbin, China; 3 National Key Laboratory for Precision Hot Processing of Metals, Harbin Institute of Technology, 150001 Harbin, China; 4 Center of Analysis and Measurement, Harbin Institute of Technology, 150001 Harbin, China; 5 National Key Laboratory of Science and Technology on Advanced Composites in Special Environments, Harbin Institute of Technology, 150001 Harbin, China

**Keywords:** Nanoscale materials, Nanoscale materials

## Abstract

We study the spin-orbit interaction of two-dimensional electron/hole gas (2DEGs/2DHGs) on quasi-2D potassium niobates (KNs) via first-principles calculations. The strong surface polarity changes the free surface states from 2DEGs to 2DHGs. The in-plane dipole maintained on 2D models leads to giant Zeeman-type spin splitting, as high as 566 meV for the (001)_c_ facet KN and 1.21 eV for the (111)_c_ facet KN. The thickness-dependent Zeeman-type spin splitting shows a linear relation with respect to 1/*r*, while the corresponding in-plane polarization quantum has a linear relation of 1/(2^0.5)with respect to a decrease in thickness. Interestingly, the 2DHGs with molecular-like orbital character is solely constituted by O 2*p* states, showing logic switchable behavior at extremely thin samples with enormous Zeeman-type splitting that can switch between insulator and conductor by opposite spin polarization.

## Introduction

Recently, two-dimensional (2D) perovskite oxides have attracted growing interest due to their exotic electronic properties. Many significant works have been reported focusing on the fabrication of these materials. Free-standing SrTiO_3_ and BiFeO_3_ ultrathin films synthesized by reactive molecular beam epitaxy have been reported^1^, providing an effective preparation route for 2D perovskite oxides with varying thicknesses, which makes it possible to develop 2D oxide electronics. The interaction of the electron spin and its momentum – spin-orbit interaction (SOI) – lays the cornerstone of manipulating the trajectories of an electron by spin^2^, leading to fascinating quantum phenomena such as the spin-Hall effect^3^, spin ballistic transport^4^, the spin-galvanic effect^5^, and topological insulators^6^. However, limited success of Rashba or Zeeman-type spin splitting has been achieved due to the challenge of breaking the spatial or time-reversal symmetry around momentum *k*, where the spin polarization of electron/hole pockets is usually symmetry-forbidden when formed around time-reversal k-points in oxide systems^7^. However, recent spin- and angle-resolved photoemission spectroscopy results show that a nontrivial magnetic moment (breaking the time-reversal symmetry) can be obtained by the formation of clusters of oxygen vacancies on the bare SrTiO_3_ surface, leading to a non-degenerate Rashba-type spin splitting at the Brillouin zone center. The Rashba-type spin splitting in perovskite oxides is a potential candidate for yielding a single Fermi contour (where the spin orientations are locked to the momenta) on the Fermi surface, which is the key ingredient for achieving the hotly pursued Majorana fermion in conjunction with its superconductivity^8–10^. Whereas, forming pure non-degenerate subbands near Fermi-level with opposite spin directions - the Zeeman-type spin splitting - would endow materials with many interesting phenomena such as a field-effect-induced spin Hall effect^7^. Specifically, the giant Zeeman-type spin splitting of 2D free electron/hole states in 2D perovskite oxides with spin-polarized half-metallicity would provide a new route for fabricating giant/tunnel magnetoresistance devices^11^. As the SOI Hamiltonian is described by ${\hat{H}}_{{\rm{so}}}=-\,{\mu }_{B}{\boldsymbol{\sigma }}\cdot {{\bf{B}}}_{{\rm{eff}}}$ (*μ*_*B*_ is the Bohr magneton, σ represents the spin Pauli matrices, and **B**_eff_ is the effective magnetic field), the direction of **B**_eff_ is principally dependent on the particle momentum **p** and electron field **E**. In a 2D system, momentum **p** is constrained in the 2D plane; thus, the direction of the electron dipole (out of plane or in plane) in a 2D system plays a decisive role in the formation of Rashba or Zeeman-type spin splitting. Introducing the magnetic momentum along the z direction of 2D oxides would give rise to Zeeman-type spin splitting.

## Results and Discussion

**Figure 1:**
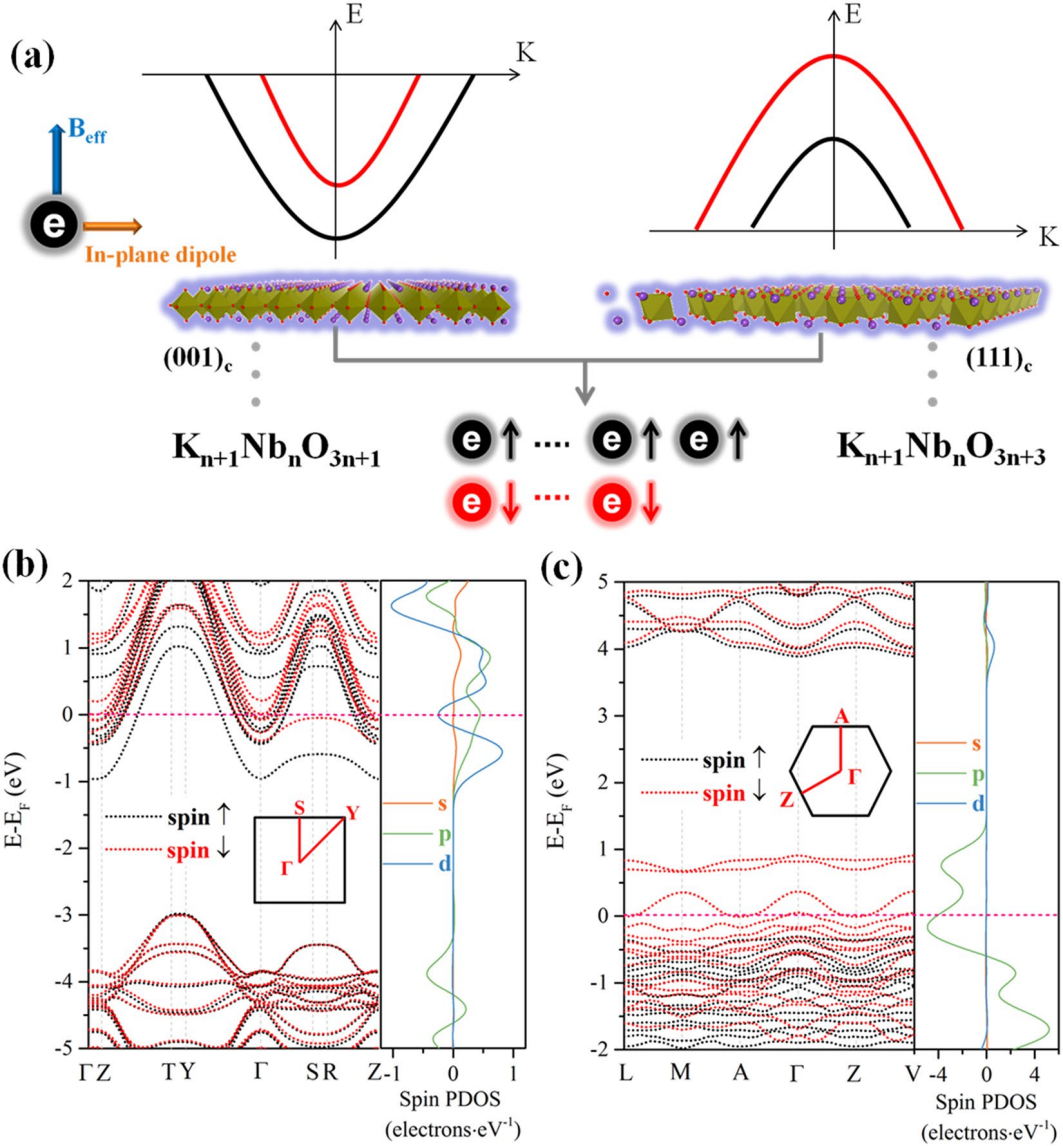
Zeeman-type SOI effect and the electronic structure of quasi-2D KNbO_3_. (**a**) Schematics representing the origin of Zeeman-type SOI on (001)_c_ and (111)_c_ facet models with 2DEGs and 2DHGs, respectively. Odd spin-up electrons are maintained on both 2D models, leading to the intrinsic magnetic order. (**b**) Calculated band structure and SPDOS of the (001)_c_ facet model with a thickness of 0.80 nm. (**c**) Calculated band structure and SPDOS of the (111)_c_ facet model with a thickness of 0.46 nm.

Here, we first propose an intrinsic Zeeman-type spin splitting based on the 2DEGs/2DHGs system of a quasi-2D perovskite niobate in which its inversion symmetry is broken by maintaining an additional dipole in its 2D plane, as depicted in Fig. 1a.

The formulas of K_n+1_Nb_n_O_3n+1_ and K_n+1_Nb_n_O_3n+3_ are theoretical 2D models used in the *ab initio* calculation, with facets of (001)_c_ and (111)_c_ (oriented by 0° and 60° tilted oxygen octahedrons, respectively). 2D crystal models are relaxed through geometry optimization according to system energy, force and stress by density functional theory (DFT, implemented in the Cambridge Serial Total Energy Package^12^) with the Perdew, Burke, and Ernzerhof (PBE) functional^13^. Both of the models are cut off by a K-O plane as the terminating surface. Hydrogen atoms are used to cap the dangling bonds of both surfaces to avoid the polar catastrophe^14^.

Dissimilar to traditional 2DEGs materials based on SrTiO_3_^15^, in which the outer-shell valence is composed of 5s^2^ for Sr and 3d^2^4s^2^ for Ti, the niobate perovskite shows an odd number of valence electrons; for instance, KNbO_3_ is composed of 4s^1^ for K and 4d^4^5s^1^ for Nb, giving rise to an out-of-plane effective magnetic momentum (*B*_eff_) on both quasi-2D models due to the breaking of time-reversal symmetry, as shown in Fig. 1a. The quantum-confined spin-up electrons construct the out-of-plane magnetic order, transforming such a band insulator into a conductor and magnets, as sketched in Fig. 1a. After hydrogen capping of their surfaces, two different crystal facet configurations were evaluated with distinct origins of conductivity. The (001)_c_ facet shows the traditional n-type free electron conduction (2DEGs), while the (111)_c_ facet maintains abnormal p-type free hole (2DHGs) conduction. The reversed behavior is ascribed to the massive polar anisotropy of the varied facets, and more than 200 meV/Å^2^ differences in surface energy (calculated by the PBE functional) can be obtained between (111)_c_ and (001)_c_. Figure 1b,c shows the calculated electronic structures of the (001)_c_ facet (0.80 nm sample) and (111)_c_ facet (0.46 nm sample) oriented quasi-2D KN, respectively. Strong spin-polarized free electron or hole states (approximately 1 eV in energy) can be observed in both cases (marked by light gray areas). Compared with the confined free states around the Fermi level (*E*_*F*_), subbands distant from *E*_F_ show small energy splitting [details can be seen in the right panels of Fig. 1b,c, which quantitively give the distribution of the spin density of states (SDOS) of both models], indicating that the spin-polarized free states are mainly contributed by 2D confined Fermi gas.

**Figure 2:**
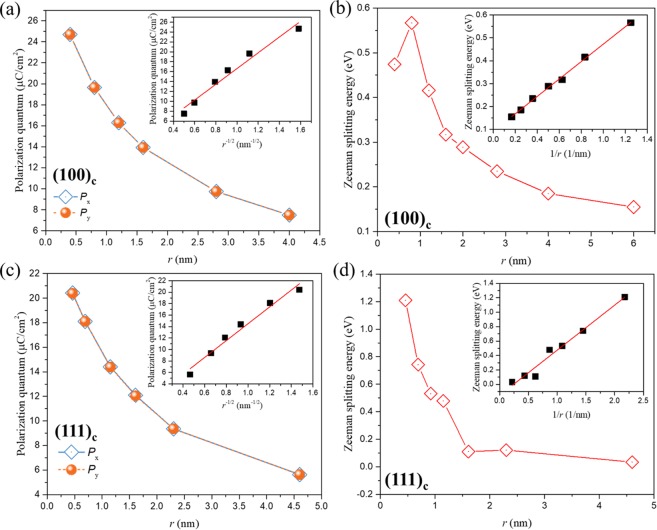
Calculated polarization and Zeeman spin splitting of 2D KN. (**a**) Polarization quantum of 0.40 nm, 0.80 nm, 1.20 nm, 1.60 nm, 2.80 nm and 4.00 nm 2D KN models of the (001)_c_ facet (inset shows the linear fitting of polarization quantum with respect to *r*^−1/2^). (**b**) The thickness dependence of the calculated Zeeman spin splitting of conduction band minimum (CBM) subbands of the (001)_c_ facet (inset shows the linear fitting of Zeeman spin splitting with respect to 1/*r*). (**c**) Polarization quantum of 0.46 nm, 0.69 nm, 0.92 nm, 1.15 nm, 2.30 nm and 4.60 nm 2D KN models of the (111)_c_ facet (inset shows the linear fitting of polarization quantum with respect to *r*^−1/2^). (**d**) The thickness dependence of the calculated Zeeman spin splitting of valence band maximum (VBM) subbands of the (111)_c_ facet (inset shows the linear fitting of Zeeman spin splitting with respect to 1/*r*).

**Figure 3:**
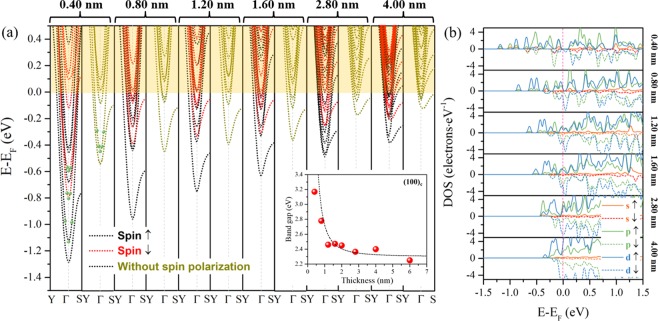
Calculated electronic structures of the (001)_c_ facet quasi-2D models. (**a**) Band structures of 0.40 nm, 0.80 nm, 1.20 nm, 1.60 nm, 2.80 nm and 4.00 nm 2D models with (black and red dotted lines) and without (cyan dotted lines) spin polarization. The green balls in the 0.40 nm subbands mark the abnormal contribution of the K 3*p* states. The black dotted line indicates the theoretical band gaps and is obtained by fitting the calculated gap values (red ball) using a 2D quantum well model. (**b**) Calculated PDOS of (001)_c_ facet models with thicknesses of 0.40 nm, 0.80 nm, 1.20 nm, 1.60 nm, 2.80 nm and 4.00 nm. Solid lines are for spin-up states, while the dashed lines are for spin-down states. The colors orange, green and blue correspond to the *s*, *p*, and *d* states, respectively.

Under Zeeman-type SOI, the Hamiltonian of an electron/hole in a 2D quantum well is described by $\hat{H}={\hat{H}}_{0}+{\hat{H}}_{c}+{\hat{H}}_{z}$, where ${\hat{H}}_{c}$ and ${\hat{H}}_{z}$ describe the contributions of the quantized well potential and the Zeeman-type SOI, respectively. Hence, their intrinsic energy dispersion can be written as $E(k)=E({k}_{0})+\frac{{\hslash }^{2}{k}^{2}}{2M}+\frac{g{\mu }_{B}B}{2}$^16^, where *M* is the effective mass-related parameter and *g* factors define the power of the Zeeman-type SOI. Treating the ${\hat{H}}_{z}$ perturbatively^17^, ${g}_{{\rm{z}}}={g}_{0}+g{\prime} {k}^{2}$, ${g}_{0}\approx 2$ is the Landè factor, and $g{\prime} {k}^{2}$ corresponds to the second-order perturbative term^18^. Considering that the origin of spin splitting is symmetry breaking in 2D KN, the in-plane polarization quantum (*P*_x,y_) along orthogonal directions is calculated with various thicknesses. ${P}_{{\rm{x}},{\rm{y}}}=\frac{|e|{R}_{i}}{\Omega }$, where $|e|$ is the electronic charge, ${R}_{i}$ is the *i*′th lattice vector, and $\Omega$ is the unit cell volume, this equation is given by the self-returned Hamiltonian of the change in polarization per unit volume for momentum *k*^19^. As shown in Fig. 2a,c, the values of *P*_x,y_ for both the (100)_c_ and (111)_c_ facet 2D KN show an evolutionary tendency of *r*^−1/2^ with respect to a decrease in thickness and show almost identical values for orthogonal x and y directions of each thickness, which means that *P*_x,y_ is isotropic in orthogonal directions of the 2D plane. The Zeeman splitting energy for an electron or hole can be described by *E*_z_ = *E*_σ↓_ − *E*_σ↑_ =${g}_{{\rm{z}}}{\mu }_{B}{B}_{{\rm{eff}}}$. With a decrease in well thickness and a small *B*_eff_, $g{\prime} {k}^{2}\gg {g}_{0}$, ${g}_{{\rm{z}}}\approx g{\prime} {k}^{2}$. Taking into account the constant effective magnetic order of $\hslash /2$ and $3\hslash /2$ for constructed (001)_c_ and (111)_c_ 2D models, respectively, they would give rise to a 1/*r* relationship of *g*_z_ with respect to the well thickness, which is verified by the result of the thickness dependence of *E*_z_ in Fig. 2b,d. In the (001)c facet case, the 2DEGs is contributed by hybridization of the Nb 4*d* and K 3*p* states. Figure 3a shows the calculated conduction bands of samples with thicknesses varying from 0.40 nm to 4.00 nm.

Comparing the spin-polarized (dark and red dotted lines) and off-spin-polarized (cyan dotted lines) bands, the Zeeman-type SOI dramatically increases the absolute value of occupied energy (almost twice in energy) of free states, although this energy also increases with respect to an increase in dimension confinement (see off-spin-polarized subbands of samples with a reduction in thickness). To determine the contribution of the Zeeman-type spin splitting to such an enhanced free state, the splitting energy is obtained by calculating the difference of the CBM with the opposite spin. Conventionally, the CBM is constituted by the *t*_*2g*_ subband of Nb 4*d*^20^; however, our results show an abnormal contribution by K 3*p* electrons to the first spin-down subband (marked by green balls in the subbands of the 0.40 nm sample in Fig. 3a), which is ascribed to the subband distortion due to the strong quantum confinement at the extremely thin sample and explains the abnormal *E*_z_ of the 0.40 nm sample (Figs. 2b and 3a). The distribution detail of these abnormal K 3*p* states can be seen at the calculated partial density of states (PDOS) of the 0.40 nm sample (Fig. 3b). Furthermore, the PDOS of samples shows that the quantized states below *E*_F_ (nondegenerate K 3*p* or Nb 4*d*) would become degenerate by increasing the 2D well width and would hybridize into the condensed 2DEGs. In addition, the *E*_z_ of other samples (0.80–4.00 nm, shown as the red diamonds in the inset of Fig. 2b) present a 1/*r* relation with respect to a decrease in thickness. This relation is different from the evolutionary tendency of energy gap *E*_g_ (red dotted line in the inset of Fig. 3a) without the perturbation of the Zeeman-type SOI, which has a quadratic relation with respect to a decrease in thickness. In a 2D square well, *E*_g_ is given by ${{\rm E}}_{g}\,=\,{{\rm E}}_{g}^{0}\,+\,\frac{{\hslash }^{2}{\pi }^{2}}{2{m}^{\ast }{r}^{2}}$^21^, where *E*_*g*_ is the calculated energy gap of 2D samples, ${{\rm E}}_{g}^{0}$ is the calculated energy gap of the bulk crystal model (${{\rm E}}_{g}^{0}\,$~ 2.3 eV, calculated by the PBE functional), *r* is the sample thickness, and ${m}^{\ast }={(\frac{1}{{m}_{e}}+\frac{1}{{m}_{h}})}^{-1}$ is the reduced effective mass. The black dotted line is obtained by fitting a best-fit line to calculate *E*_g_ values of samples with varied thicknesses, where the fitting result of m^*^ ~ 0.9 is obtained for such an indirect band gap. Taking into account that the initialized spin momentum is $\hslash /2$ in (001)_c_ facet system, the calculated maximum intrinsic *E*_Z-e_ of 566 meV can be obtained for a 0.80 nm sample, twice the largest values reported for either the Rashba or Zeeman SOI systems (approximately 150 ~ 300 meV)^7,22^.

**Figure 4:**
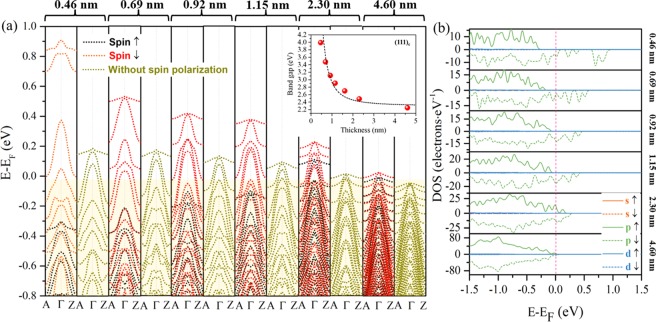
Calculated electronic structures of the (111)_c_ facet quasi-2D models. (**a**) Band structures of 0.46 nm, 0.69 nm, 0.92 nm, 1.15 nm, 2.30 nm and 4.60 nm 2D models with (black and red dotted lines) and without (cyan dotted lines) spin polarization. The inset shows the thickness dependence of the calculated energy gap. The black dotted line indicates the theoretical band gaps and is obtained by fitting the calculated gap values using a 2D quantum well model. (**b**) Calculated PDOS of (111)_c_ facet models with thicknesses of 0.46 nm, 0.69 nm, 0.92 nm, 1.15 nm, 2.30 nm and 4.60 nm. Solid lines are for spin-up states, while the dashed lines are for spin-down states. The colors orange, green and blue correspond to the *s*, *p*, and *d* states, respectively, but only *p* states are observed in this case.

By tilting the angle of the oxygen octahedron to 60°, the free states switch from CBM to the VBM. The hole conductance is merely contributed by the O 2*p* states and shows 100% spin polarization around *E*_F_ when the thickness is smaller than 1.15 nm (see Fig. 4a,b).

This phenomenon is of critical importance for realizing the application of spin-logic-circuit by using perovskite oxides, where a spin-up state leads to an insulator (presenting as |0), while the spin-down state gives rise to a conductor (presenting as |1). Compared with 2DEGs on the (001)_c_ facet KN, the 2DHGs on the (111)_c_ facet shows a stronger thickness dependence, regardless of the purely quantum confined 2DHGs that are insensitive to the spin polarization [see the cyan dotted line marking subbands of 0.46 nm to 4.60 nm samples in Fig. 4a, where the strong confined potential induced splitting of one heavy hole and two light hole subbands (0.46 nm sample), gradually becoming degenerate and decreasing to the *E*_F_ (4.60 nm sample) at a high symmetric Γ point]. The size dependence of the Zeeman-type SOI leads to a much stronger enhancement of the occupied energy of free holes, almost 5 times that of the off-spin polarized counterpart, compared to the VBM of the 0.46 nm sample in Fig. 4a. The calculated *E*_Z-h_ values of samples with thicknesses varying from 0.46 nm to 4.60 nm show a 1/*r* dependence with respect to a decrease in well width (Figs. 2d and 4a). The confined energy gap shows a quadratic relation with respect to a decrease in thickness; it is comparable to the fitted dotted line from each energy gap (m* ~ 0.6) without regard to spin polarization. The slight difference in the fitting m^*^ between the (001)_c_ and (111)_c_ facet KN might be ascribed to the transition of the band gap from indirect to direct, which is induced by the 2D symmetry change (see Fig. 1b,c). The maximum *E*_Z-h_ of ~1.21 eV is obtained for the 0.46 nm sample, which is 36 times that of the 4.60 nm sample (*E*_Z-h_ ~ 33 meV). Furthermore, the *B*_eff_ on energy dispersion is remarkably different for 2D holes than for electrons, leading to a different *E*_Z-h_ at various *k* points (samples of thickness smaller than 1 nm in Fig. 4a). The different orbital angular momenta of L = 1 for the free holes (O 2*p*) and L = 2 for the free electrons (Nb 4*d*), in conjunction with the strong lattice distortion induced by strong quantum confinement, would contribute to the momentum dependent behavior of *E*_Z-h_ It is similar to the case of *k* dependent *g* factor of holes in GaAs 2D quantum well, which the effective mass *m*_±_ for spin splitting subbands of heavy holes diverge further with an increase in *B*^23^.

In conclusion, our theoretical calculations show that the in-plane dipole can be maintained in both the (001)_c_ and (111)_c_ facet-oriented quasi-2D KN models. The constructed 2D model with time-reversal symmetry breaking along the z direction gives rise to an effective magnetic moment out of the 2D plane. In addition to the thickness dependent in-plane dipole, giant Zeeman-type spin splitting are observed. These free electrons and holes are susceptible to the Zeeman-type SOI, and the maximum *E*_z_ of 566 meV for 2DEGs and 1.21 eV for 2DHGs can be obtained. Furthermore, these values all follow a 1/*r* rule with respect to the dimension reduction. Interestingly, taking advantage of giant Zeeman-type spin splitting, 100% spin-polarized O 2*p* states can be maintained near *E*_F_, which provides a new route toward the application of all-oxide spintronic devices in on-chip logic circuits within an extreme scale. Experimentally, we have developed a novel synthesis method that allows the topotactic growth of freestanding perovskite niobates into specific 2D facets, taking the first step toward the realization of spin computing in the near future.

## Methods

The first-principles calculations are performed with the Cambridge Serial Total Energy Package (known as CASTEP). A bulk KNbO_3_ crystal is established first and then cut off by a K-O plane as the terminating surface to build 2D crystal slabs with different crystal surfaces exposed. There should be a vacuum spacing layer with a value of 1 nm between neighboring repeat units of 2D crystal slabs. Hydrogen atoms are set to balance the surface charge of oxygen bonds^14^. Geometry optimization based on DFT with the Perdew, Burke, and Ernzerhof functional is performed to relax the crystal models^13^. The plane-wave cut off energy is set to 340 eV, and pseudopotentials that describe the ionic core are set to ultrasoft pseudopotentials. The *k*-point sampling grid for bulk KNbO_3_ is 4 × 4 × 2, while it is 3 × 3 × 1 for 2D crystal slabs.

The in-plane polarization properties are calculated by the Perdew, Burke, and Ernzerhof functional available in commercial software Quantum ATK with PseudoDojo pseudopotential. The *k*-point sampling grid for 2D crystal slabs is 6 × 6 × 1 for 2D crystal slabs. All of the 2D slabs are fully relaxed.

## Data Availability

The datasets generated during and/or analyzed during the current study are available from the corresponding author on reasonable request.
